# Supplementary figures and images for: Systematic comparison of modeling fidelity levels and parameter inference settings applied to negative feedback gene regulation
Source: PLoS Comput Biol. 2022 Dec 15;18(12):e1010683. doi: 10.1371/journal.pcbi.1010683 (PMC9799300; doi:10.1371/journal.pcbi.1010683)

# Model Utility based on the Kolmogorov distance

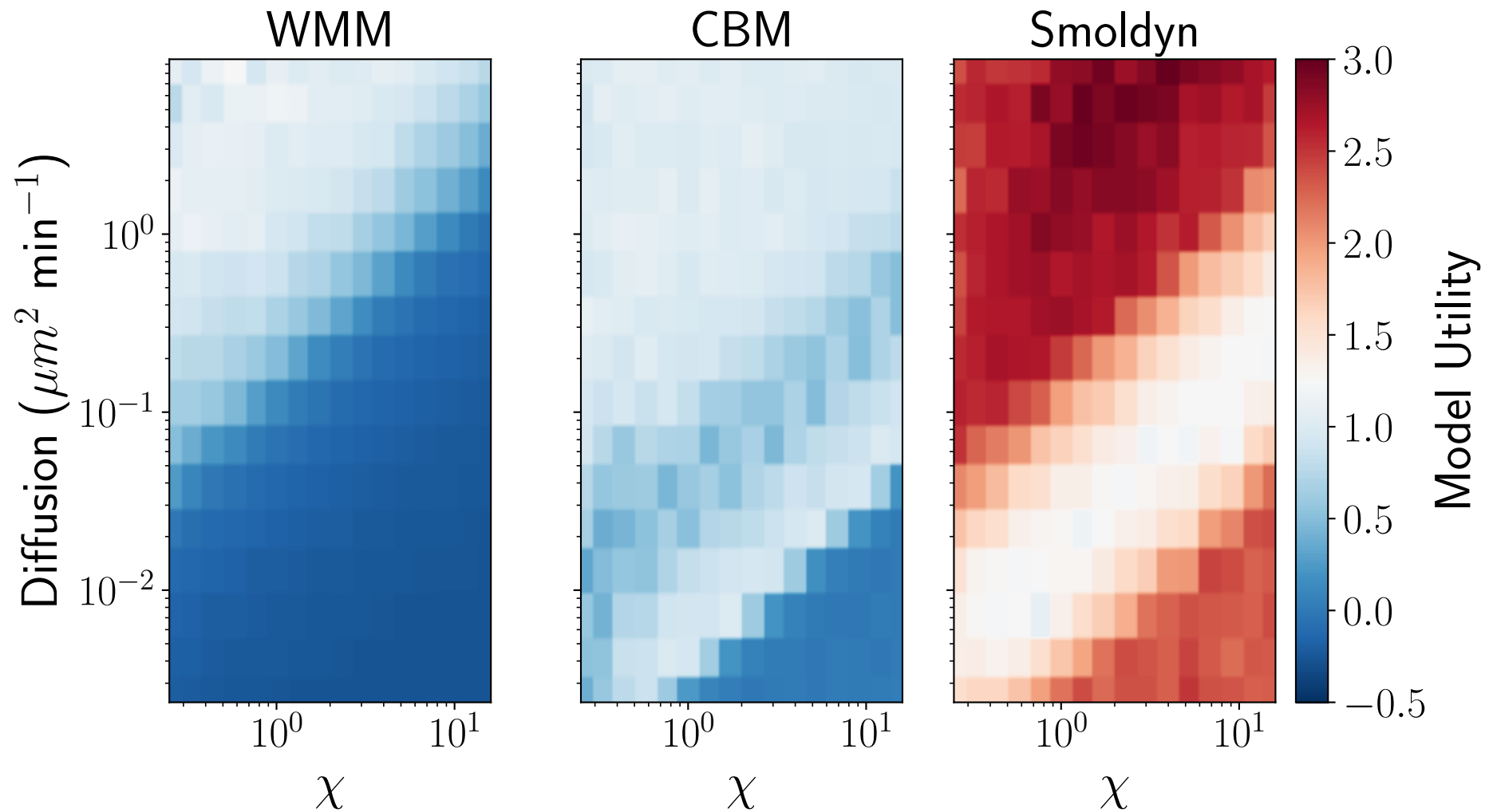

Supplement: S1 Fig — The y-axes shows how the model utility varies with the diffusion constant and x-axes shows how it varies with the reactivity constant. All axes are displayed in log scale and so too is the colour bar. Overall, the model utilities do not correlate with the error estimates presented in Fig 4. (PDF) [file pcbi.1010683.s001.pdf]

## WMM

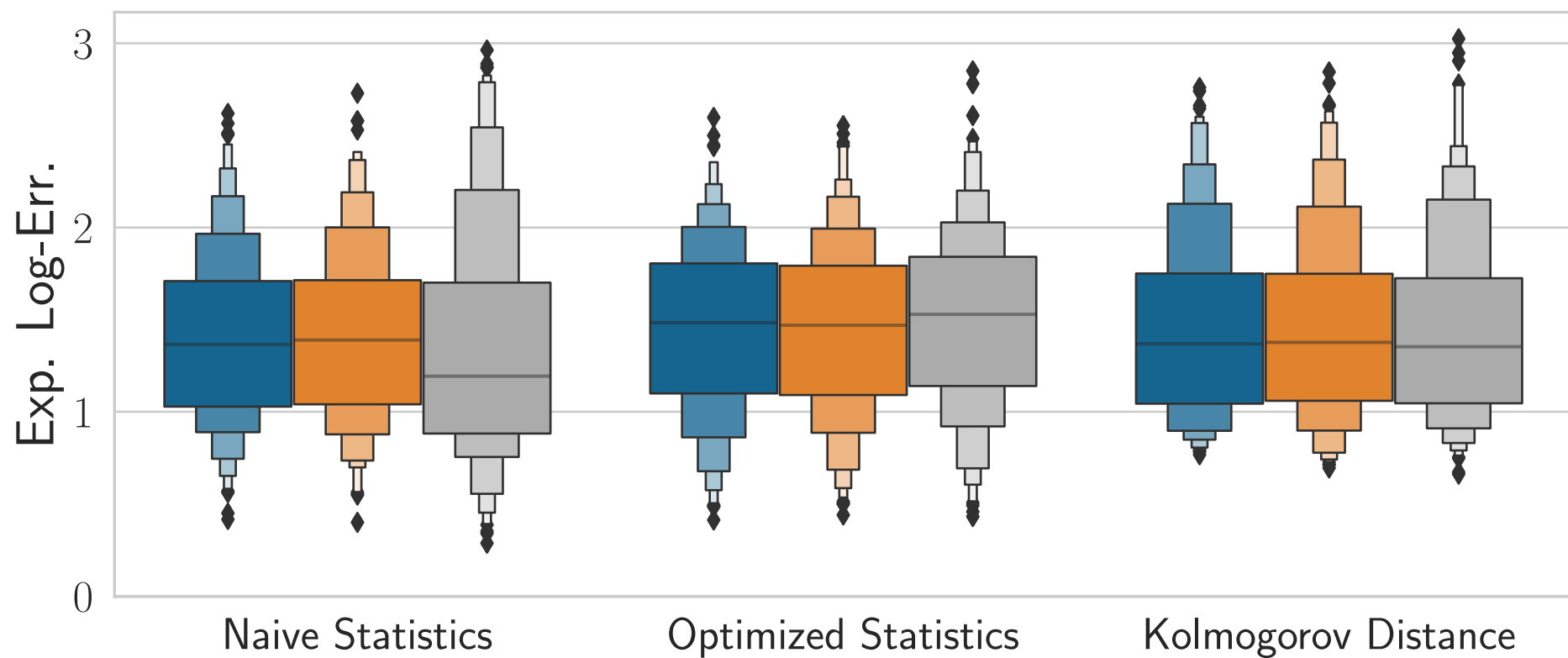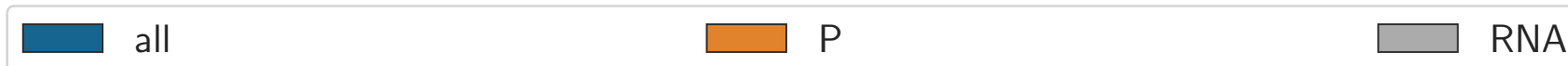

## CBM

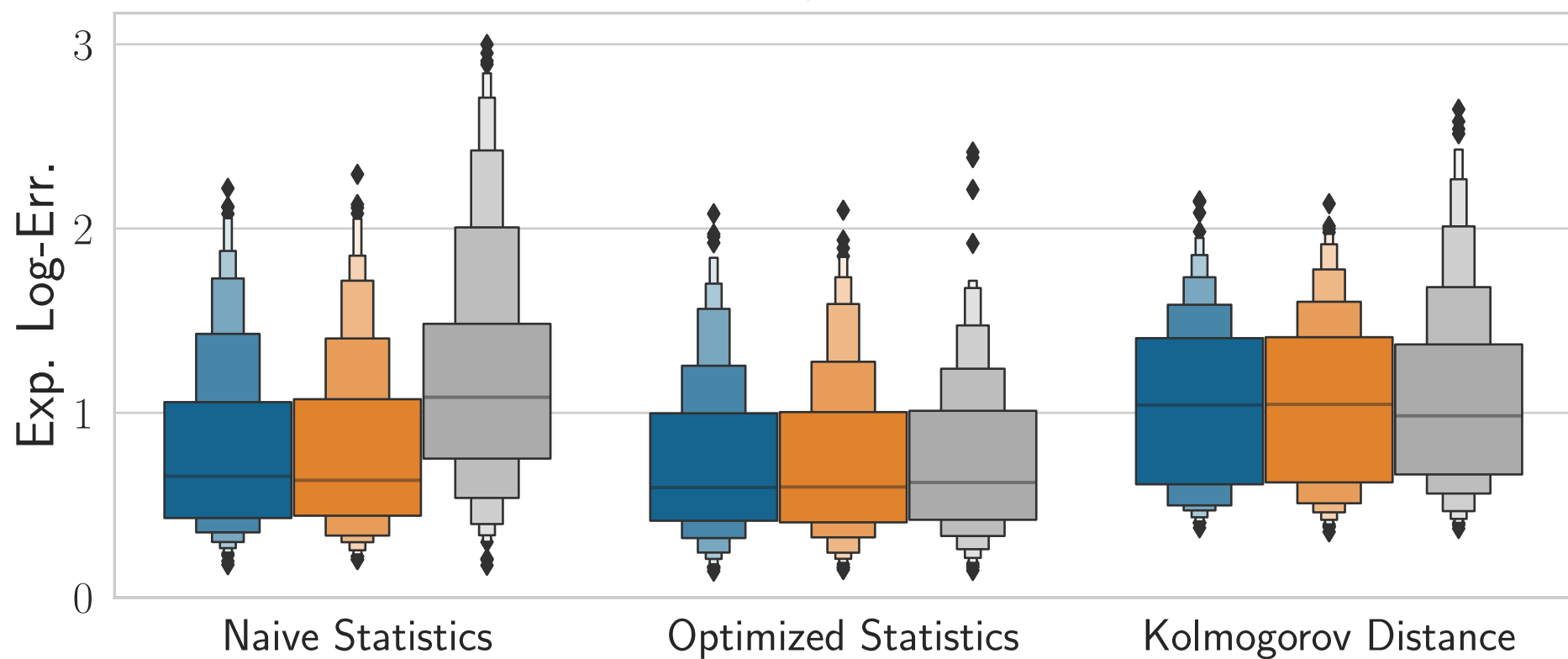

Supplement: S2 Fig — Overall no big difference can be observed, contrary to the case where the Smoldyn was used (see Fig 7). (PDF) [file pcbi.1010683.s002.pdf]

# Optimized Statistics

WMM

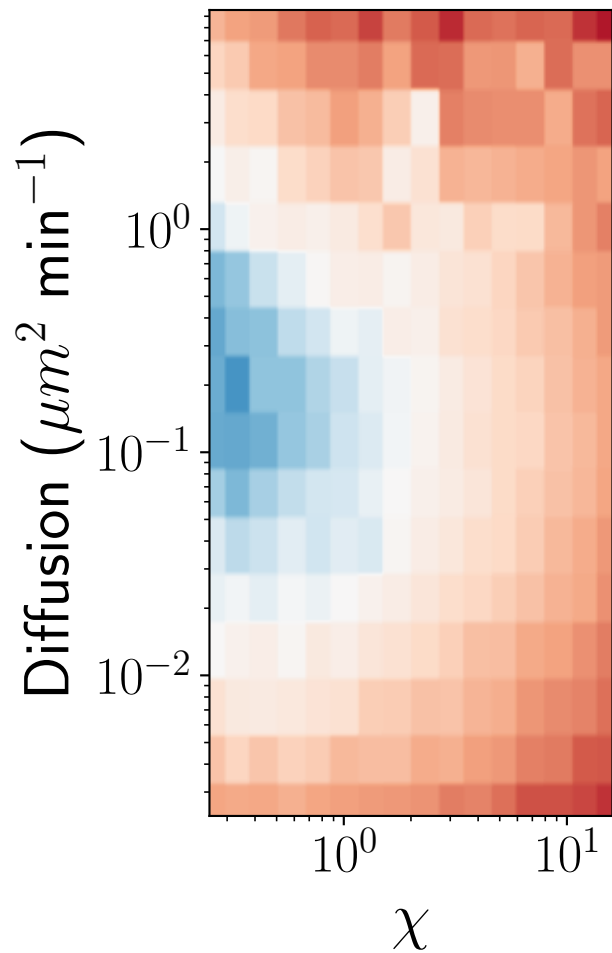

CBM

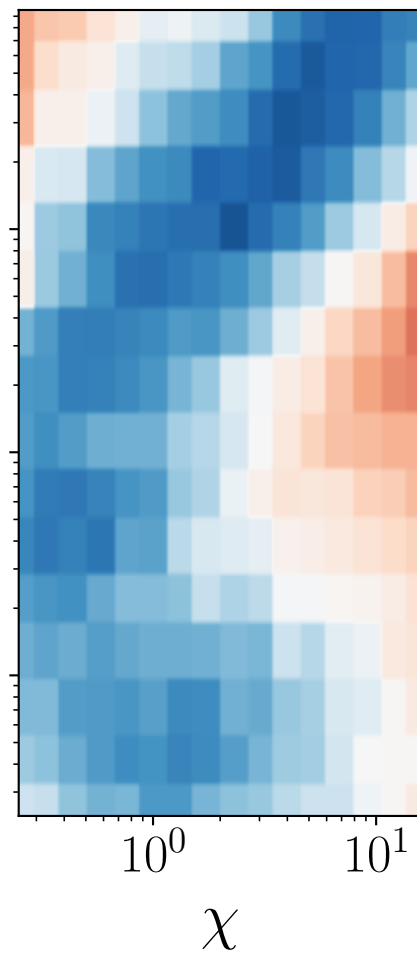

Smoldyn

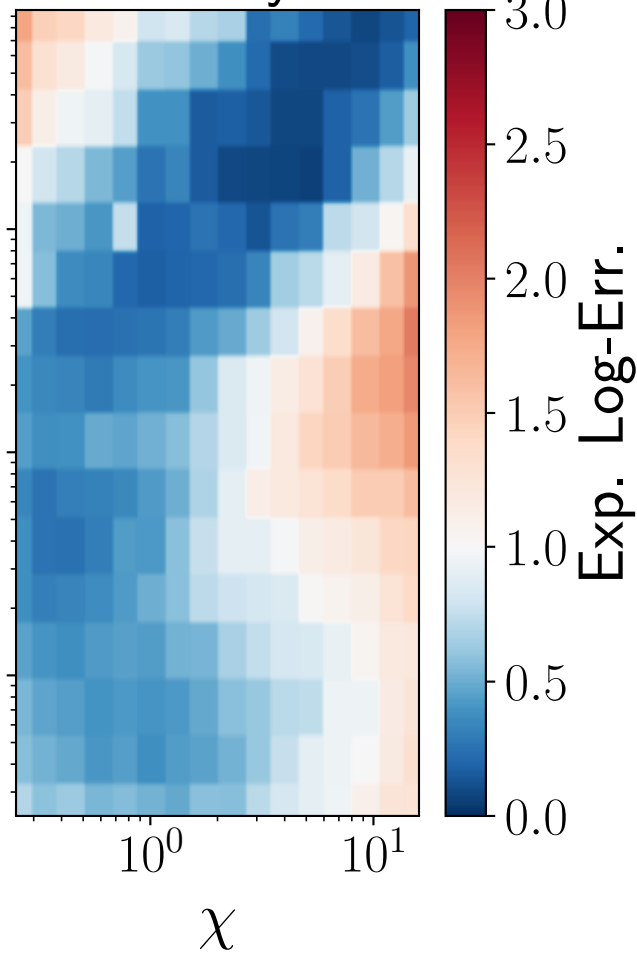

Supplement: S3 Fig — The y-axes shows how the expected log-error varies with the diffusion constant and x-axes shows how it varies with the reactivity constant. The error is slightly lower than when only basic summary statistics are used. (PDF) [file pcbi.1010683.s003.pdf]

# Distance metrics

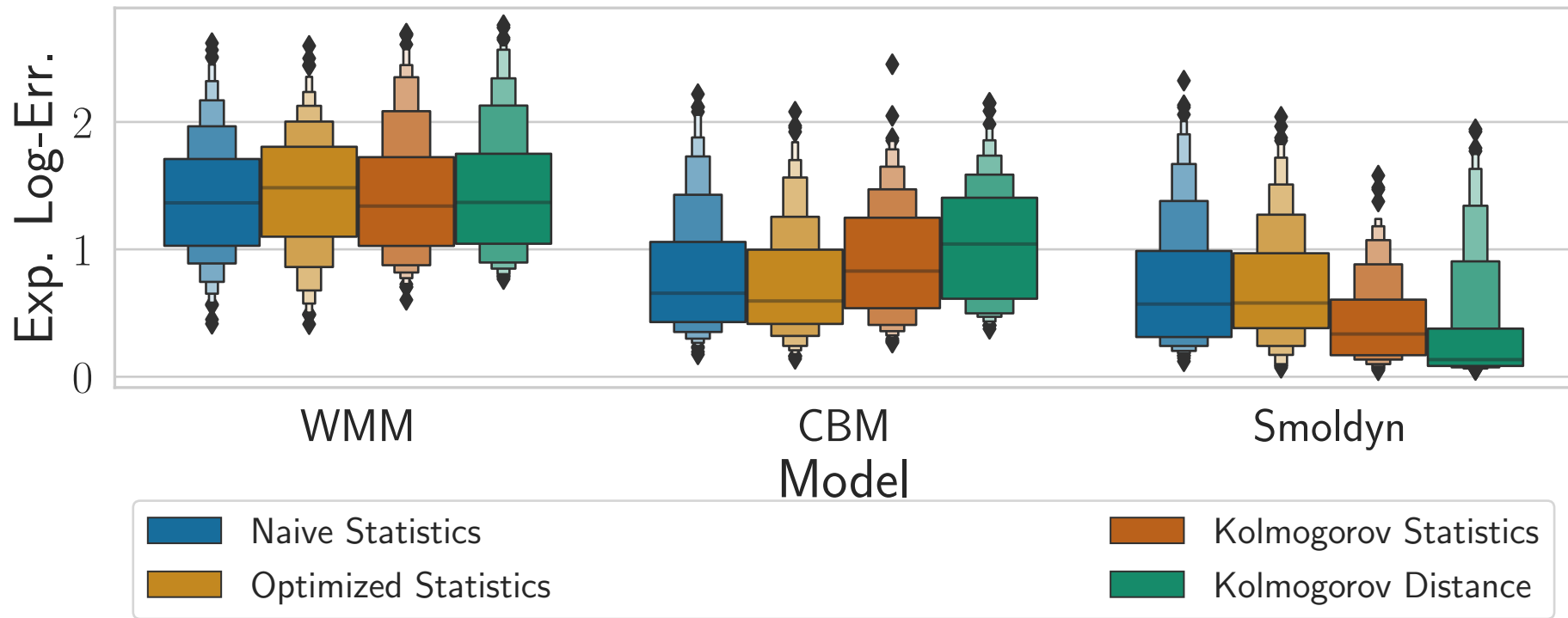

Supplement: S4 Fig — When comparing simulated and true data via summary statistics for datasets with multiple trajectories the most straightforward and the most common way is to compute the statistic for each trajectory and then compare the expected values for true and simulated data. This is what is done in the main manuscript when using summary statistics. As a more elaborate alternative we can also compare the summary statistics using the Kolmogorov distance (Kolmogorov statistics). This entails computing the histogram CDF for the statistic at each timepoint, then taking the Kolmogorov distance between true and observed data. As can be seen, this approach is advantageous when using the Smoldyn simulator, however it does not lead to as low errors as directly comparing the copy numbers as done in the main manuscript (Kolmogorov distance). For the CBM model, however, taking a distribution measure leads to higher error. (PDF) [file pcbi.1010683.s004.pdf]

## Naive Statistics

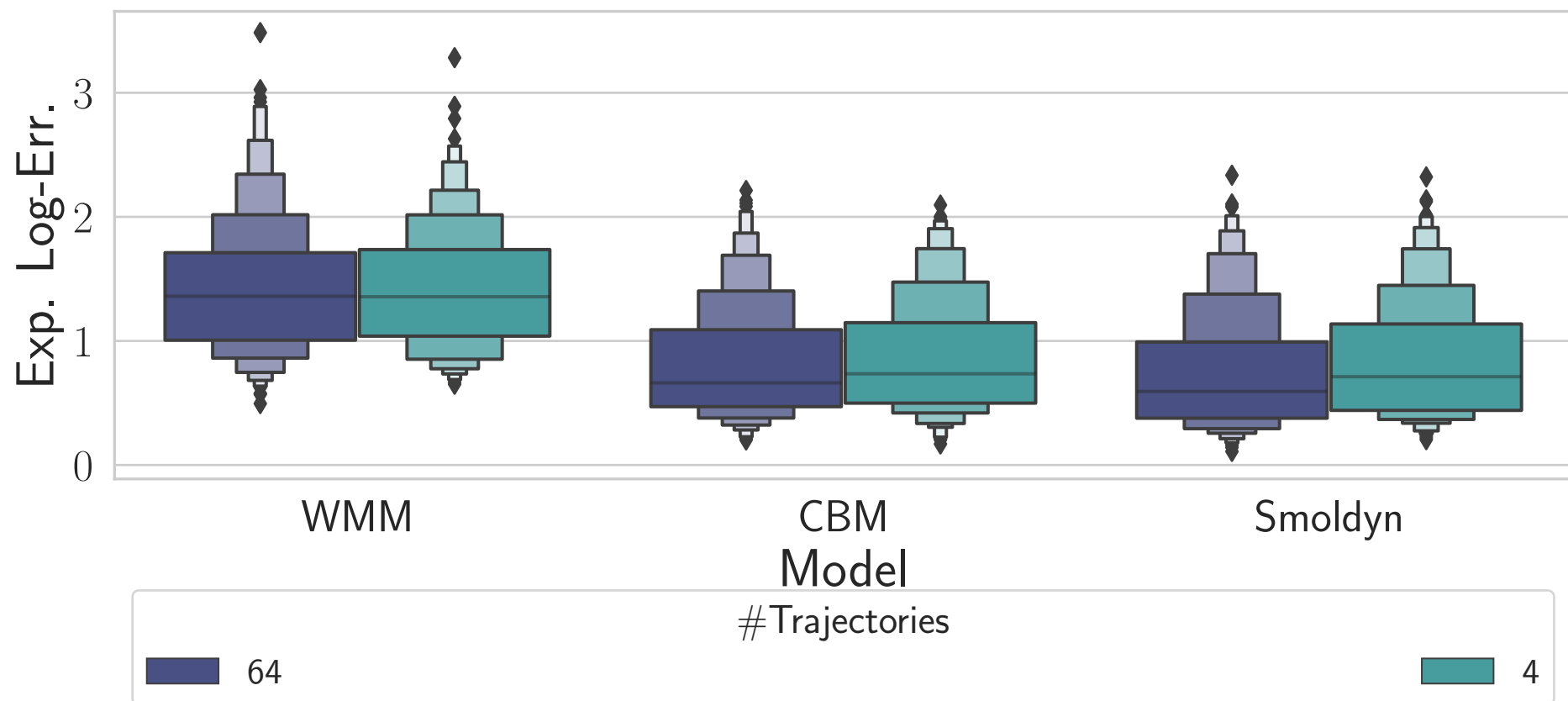

## Kolmogorov Distance

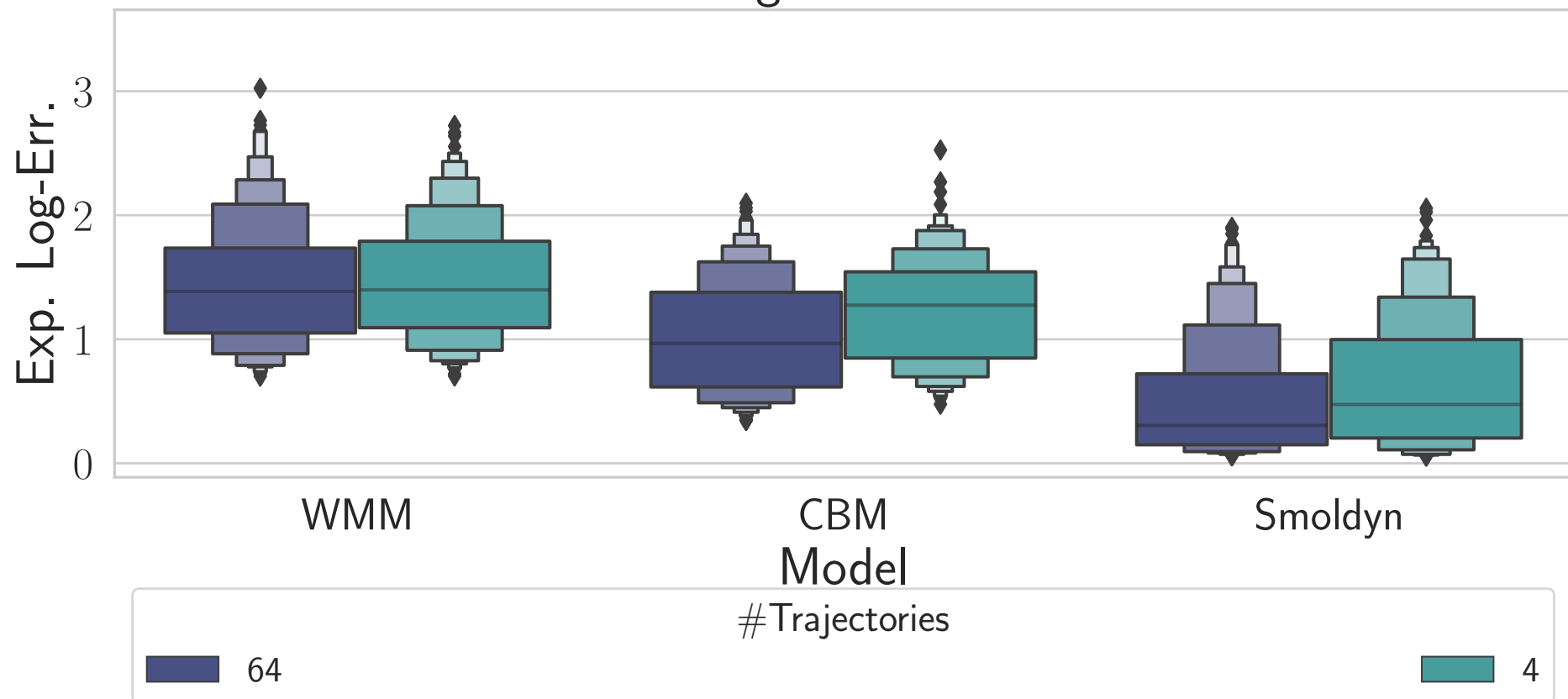

Supplement: S5 Fig — In general, increasing the granularity of the data has a greater effect when using the Kolmogorov distance, where the accuracy improves for both the CBM and Smoldyn. (PDF) [file pcbi.1010683.s005.pdf]

# Naive Statistics

## WMM

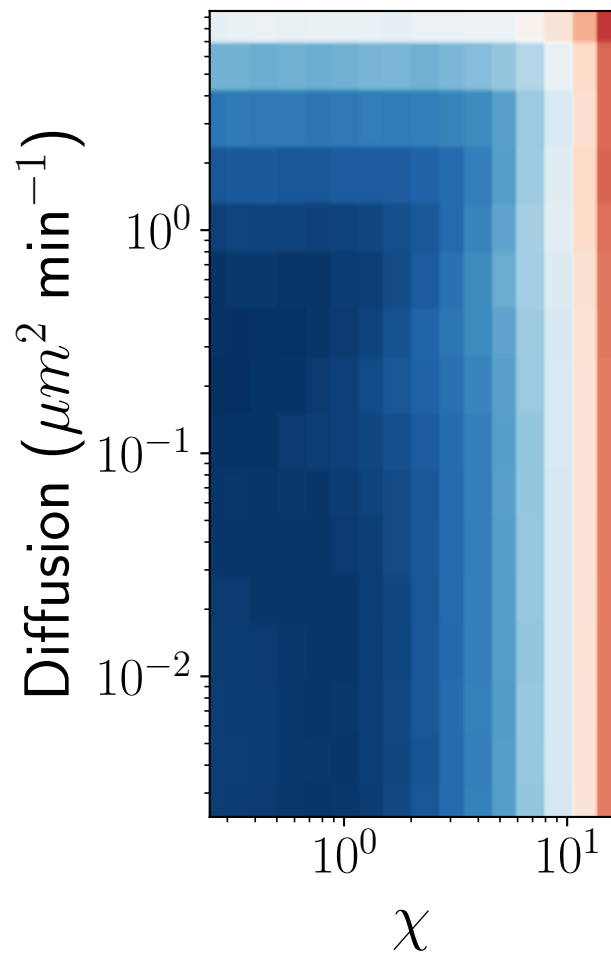

## CBM

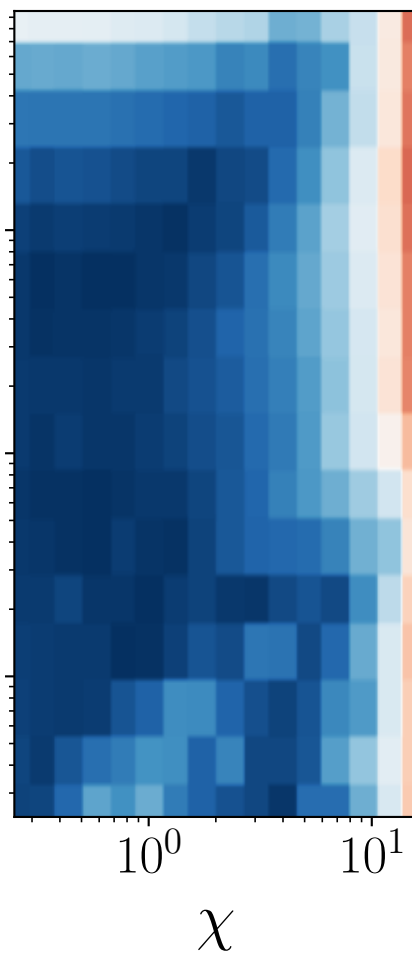

## Smoldyn

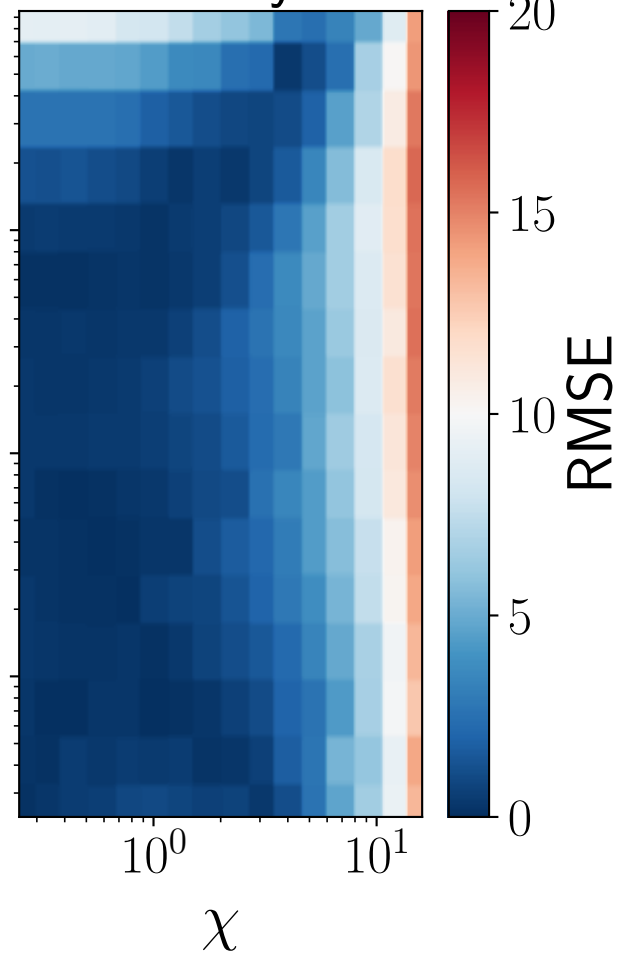

Supplement: S6 Fig — While there are quantitative differences, the same general trend persists. (PDF) [file pcbi.1010683.s006.pdf]
