# Supplementary material for: Systematic comparison of modeling fidelity levels and parameter inference settings applied to negative feedback gene regulation
Source: PLoS Comput Biol. 2022 Dec 15;18(12):e1010683. doi: 10.1371/journal.pcbi.1010683 (PMC9799300; doi:10.1371/journal.pcbi.1010683)
Supplement: S1 Text — Explanation of each of the summary statistics used. (PDF) [file pcbi.1010683.s007.pdf]

Summary Statistic Definitions

The TSFRESH Python library was used to extract time series features as summary statistics, and implements the following definition of the candidate summary statistics.

- sum of values: the sum over the time series values.
- absolute energy: the absolute energy E of a time series x is defined as, E = $\sum_{i=1,\ldots,n} x_i^2$.
- mean absolute change: the mean over absolute differences between subsequent time series values, defined as, $1\frac{}{n-1\sum_{i=1,\ldots,n-1}|x_{i+1}-x_i|.}$
- mean change: the mean over differences between subsequent time series values, defined as, $1\frac{}{n-1\sum_{i=1,\ldots,n-1}x_{i+1}-x_i.}$
- median: the median over time series values.
- mean: the mean over time series values.
- length: the length of the given time series.
- standard deviation: the standard deviation over the time series values.
- skewness: the sample skewness of the time series x, calculated using the adjusted Fisher-Pearson standardized moment coefficient G1.
- kurtosis: the kurtosis of the time series x, calculated using the adjusted Fisher-Pearson standardized moment coefficient G2.
- longest strike below mean: the length of the longest consecutive subsequence in x that is smaller than the mean of x.
- longest strike above mean: the length of the longest consecutive subsequence in x that is larger than the mean of x.
- last location of maximum: the last location of the maximum value over x, calculated relative to the length of x.
- first location of maximum: the firt location of the maximum value over x, calculated relative to the length of x.
- last location of minimum: the last location of the minimal value over x, calculated relative to the length of x.
- first location of minimum: the first location of the minimal value over x, calculated relative to the length of x.
- maximum: the maximum value over the time series x.
- minimum: the minimum value over the time series x.
